# Lifetime Practice and Intention to Use Contraception After Induced Abortion Among Serbian Women in Belgrade

**DOI:** 10.3390/medicina60121944

**Published:** 2024-11-26

**Authors:** Tatjana Gazibara, Jovan Bila, Lidija Tulic, Natasa Maksimovic, Jadranka Maksimovic, Jelena Stojnic, Dragana Plavsa, Maja Miloradovic, Milos Radovic, Katarina Maksimovic, Jelena Dotlic

**Affiliations:** 1 Institute of Epidemiology, Faculty of Medicine, University of Belgrade, 11000 Belgrade, Serbia; tatjanagazibara@yahoo.com (T.G.);; 2 Clinic for Obstetrics and Gynecology, University Clinical Center of Serbia, 11000 Belgrade, Serbia; 3 Faculty of Medicine, University of Belgrade, 11000 Belgrade, Serbia; 4 Institute of Public Health of Serbia “Dr Milan Jovanovic Batut”, 11211 Belgrade, Serbia; 5 Institute of Hygiene and Medical Ecology, Faculty of Medicine, University of Belgrade, 11000 Belgrade, Serbia

**Keywords:** induced abortion, contraception, intention, women

## Abstract

*Background and Objectives*: The issue of high rates of abortion among Serbian women has been previously highlighted, yet its social underpinnings are still not clear. The aim of this study was to investigate the lifetime use of and intention to use contraception among women after having an abortion. *Materials and Methods*: A cross-sectional study was carried out from 2022 to 2024 at the Clinic for Obstetrics and Gynecology, University Clinical Center of Serbia. The study participants were women who underwent induced abortions at the clinic. They filled in an anonymous questionnaire examining their demographic data, life-style and habits, medical history, lifetime use of contraception and intention to use contraception after their abortion. *Results*: A total of 433 women aged 16 to 49 years (mean age 32.0 years) participated in the study. In our sample, 81.1% of women had ever used contraception, with condoms being the most common, while 18.9% never used any contraception. Around 70% of women expressed the intention to use contraception post-abortion. Women who were of Serbian ethnicity, who had a higher level of education, who had no chronic illnesses and who already had multiple children were more likely to ever use contraception. Being of Serbian ethnicity, having higher education level and chronic illnesses and not smoking were associated with the intention to use contraception post-abortion. *Conclusions*: Most women who had abortions used contraception at least occasionally during their reproductive life and had the intention to start using it again. Therefore, women need to be continuously reminded by their gynecologists of contraception possibilities.

## 1. Introduction

Access to safe abortion care has been defined as a basic human right [1,2], despite having political, moral and religious controversies attached to it [3]. More than 70 million women worldwide seek the voluntary termination of pregnancy each year [2]. However, there are remarkable variations between geographic regions in the prevalence of seeking abortion. For example, in the European region, women in eastern Europe seek abortion 2.5 times more frequently compared to women in western/northern Europe (41 vs. 18 per 1000 women) [3]. Although safe abortion care is provided in almost all European countries, as well as a wide variety of contraception methods, these discrepancies in abortion rates suggest that the underlying reasons to seek abortion may be a more complex issue.

In Serbia, a country in eastern Europe, the first legal regulations on abortion rights were introduced in 1929, and since 1974, the right to abortion has been a part of the constitution. At the same time, a wide array of contraceptive methods have been accessible and can be bought in public and private pharmacies and convenience stores. However, few young women use oral contraceptive pills [4] and condoms [5]. Counseling on contraception post-abortion has been in place for adolescents [6], but no other initiatives in primary health centers and schools exist [7]. The issue of high rates of abortion among Serbian women has been discussed in the literature [8,9], yet its social underpinnings are still not clear.

Although Serbia has a relatively small population of just over 6 million people, more than 1.5 million women are in need of family planning to avoid unwanted pregnancies and abortion care. Thus, the knowledge of contraception and its use are pressing issues relevant for women’s health in Serbia. Unveiling the practices and intention to use contraception among women who have had an abortion could provide a clearer picture of what women need to improve the prevention of abortions and increase the use of contraceptives to inform policy makers and other stakeholders.

The aim of this study was to investigate the lifetime use of and the intention to use contraception among women who had had an induced abortion due to unwanted pregnancy in Belgrade, Serbia.

## 2. Materials and Methods

### 2.1. Setting

A cross-sectional study was conducted at the Clinic for Obstetrics and Gynecology, University Clinical Center of Serbia, which is one of five publicly financed tertiary health centers in Belgrade, where women can receive comprehensive abortion care. Over the past decade, on average, around 200 induced abortions were performed each year at our clinic.

The termination of pregnancy on demand is granted by law up to 10 gestational weeks. However, induced abortions are not covered by health insurance (either public or private) and patients are mandated to pay out of pocket for the procedure (costing around EUR 150 to 200 in public and up to EUR 500 in private clinics). The age of maturity in Serbia is 18 years, but girls who are 16 and 17 years old do not require a parent or a guardian when seeking induced abortion.

At our clinic, abortions are only performed surgically by vacuum aspiration and the curettage of the uterine cavity at the Department for Family Planning and Minor Gynecological Interventions. Women are admitted for a short stay at the hospital. After the intervention, women are observed for two hours at the department to ensure that there are no immediate complications. If no complications arise, women are discharged on the same day with a suggested therapy and a recommendation to receive a gynecological check-up seven days after the intervention. Finally, if there are no issues with recovery after induced abortion, women are advised to contact their chosen gynecologist at the Primary Health Center to discuss the potential use of contraception.

### 2.2. Study Participants

The study sample was formed using a non-probability convenience sampling methodology, i.e., including all women who fulfilled the inclusion criteria. The sample size was calculated using an online calculator. The elements needed to calculate the sample size were the estimated size of the female population in Belgrade aged 18 to 49 according to the last census (about 368,620 women) [10], the estimated prevalence of abortions (25%) [11], an alpha error probability level of 0.05 and a 95% confidence interval. The minimum sample size was 288 women. Consequently, the study included all women who underwent induced abortions at our clinic from 1 January 2022 to 31 July 2024 (the response rate was 86.2%).

Women were approached at the department, from Monday to Friday, by two researchers (study authors) who administered the questionnaires. The inclusion criteria were as follows: being older than 15 years (the minimum age for having an abortion without the presence of parents), speaking the Serbian language and providing written consent for participation. Women who seek induced abortion at our referral center come from various socio-economic groups and educational backgrounds. Thus, the study sample likely represents the female population of reproductive age in Belgrade. The Ethics Committee of the University Clinical Center of Serbia approved this study (No 1141/1).

### 2.3. Data Collection

All data were collected using an anonymous questionnaire. The questionnaire items and their responses were formed according to the available literature [12,13] as well as the results of our pilot qualitative study that applied a structured in-depth interview on 11 women to investigate decisions and behavior related to induced pregnancy abortions.

Selection bias was minimized as the study was conducted at the referral center where women of diverse backgrounds come to have induced abortions. Information bias was minimized by including a large number of items in the questionnaire. Moreover, each woman was able to fill in the questionnaire on her own in a private room. The confounding factors were minimized by including a diverse set of questions regarding medical history and socio-demographic variables.

Finally, the following information was included: socio-demographic characteristics (age, ethnicity, religion, level of education, employment, monthly salary and relationship status), life-style and habits (regular weekly recreation for at least 2 h, the physical aspects of professional activity classified according to the Center for Disease Control and Prevention as vigorous, moderate and sedentary, smoking cigarettes and drinking alcohol), general medical and gynecological history (menstrual cycle, parity, having previous cesarean sections, having previous abortions and having gynecological and/or other chronic illnesses).

We asked the women if they had ever used any contraception method and which one was their method of choice (a male condom, an intrauterine device (IUD), oral hormonal contraception (the pill), postcoital contraception, interrupted coitus, counting fertile days, other contraception methods such as other barrier methods (a diaphragm, cervical cap, or a female condom) and chemical methods (spermicides)). The women were also asked to self-assess how they felt regarding their level of knowledge about contraception (good/adequately informed vs. poor/not adequately informed about contraception methods). Finally, we explored the intention of the investigated women to start using contraception after their induced abortion. Answers were categorized in a binary way as “yes I have the intention” vs. “no I do not have the intention” to start using contraception.

After agreeing to participate and providing signed informed consent, the women filled in the questionnaire independently, while the investigators were at their disposal for clarifications, if needed.

### 2.4. Data Analysis

The obtained data were analyzed using SPSS for Windows, version 21. The statistical significance level was set at *p* < 0.05. The distribution of the sample data were assessed using the Kolmogorov–Smirnov normality test. Methods of descriptive statistics (means, standard deviations and relative numbers, i.e., percents) were used to portray the study population. The Kruscal–Wallis χ^2^ test was applied to assess the differences in the frequency of the general and medical characteristics of the examined women. Spearman’s correlation was performed to investigate the associations of use, knowledge and attitudes regarding contraception and types of contraception methods that the investigated women had used at least for some time during their life.

Binary regression modeling was performed to identify factors associated with the knowledge of contraception, lifetime use of contraception and intention to use contraception after their abortion. In the regression models, the outcome measures were defined, but no specific exposure was tested. The set of potential covariates, i.e., predictors, was defined based on previous studies [12,13]. The independent variables for each analysis were all the examined socio-demographic, life-style and medical characteristics of the investigated women. In the first model, the outcome variable (dependent variable) was self-assessed knowledge regarding contraception, classified as “good” vs. “poor”. In the second model, the dependent variable was the lifetime use of contraception, classified as “yes” vs. “no” (i.e., ever vs. never used contraceptive methods). In the third model, the dependent variable was the future intention for contraception use, classified as “yes” vs. “no”.

## 3. Results

### 3.1. Study Sample

**Figure 1:**
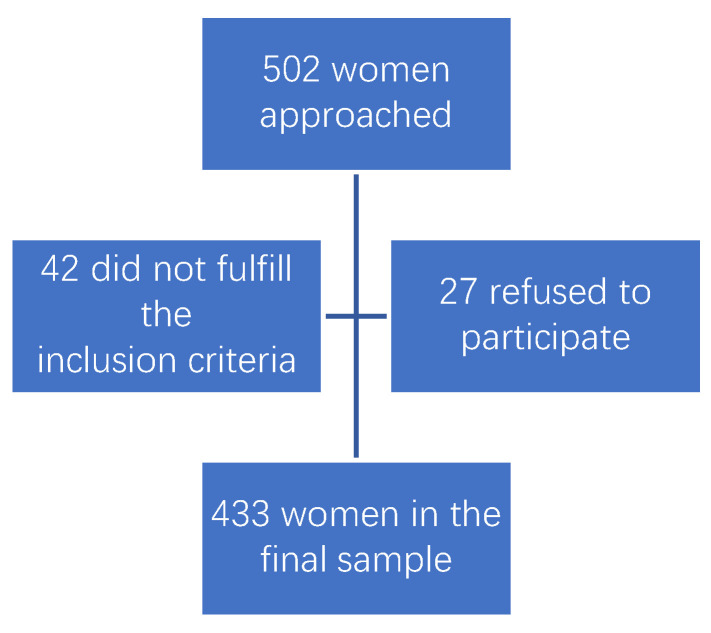
Sampling flow chart.

This study included 433 women (Figure 1) who were, on average, 32.01 ±6.68 years old (range: 16 to 49 years). The general and medical characteristics of the investigated women are presented in Table 1. The majority of the investigated women were of a Serbian ethnicity and Orthodox Christian religion. They mostly had a secondary level of education (62.1%) and were employed (58.9%). The mean salary of the investigated women was EUR 754.24 ± 34.51 (range 140 to 3000 euro), which measured up to the Serbian average.

Few women (19.4%) practiced regular recreation, and they generally considered their professional activity average. About 40% of women were smokers and drank alcohol, but most of them were not heavy smokers and drank alcohol rarely. A small proportion of the women from our sample (6.5%) had chronic illnesses, but 25% had some gynecological conditions.

More than 75% of women lived with a partner, while less than 10% of women were not in a steady relationship. The majority of women had had previous pregnancies that ended in a natural vaginal birth. They had up to five children (in average 1.5 ± 1.1). Moreover, 55.7% of women had already had a previous abortion.

### 3.2. Knowledge of Contraception

Significantly more investigated women assessed their knowledge regarding contraception as good (n = 268; 61.9%). However, almost 40% of women (n = 165) felt that they were inadequately informed about different measures of contraception accessible in Serbia.

### 3.3. Lifetime Contraception Use

In our sample, 81.1% (n = 351) of women had used some method of contraception at least for some time during their reproductive life, while 18.9% had never used any method of contraception. The condom was the most frequently used (70.4%), while other barrier methods and spermicides were the least frequently used (0.7%) contraceptives (Table 2).

### 3.4. Intention to Use Contraception in the Future

The majority of women from our sample had the intention to start with contraception post-abortion (n = 303; 70%). Still, 30% of women showed no interest in contraception even though they just had an induced abortion due to unwanted pregnancy.

### 3.5. Differences Between Women

Significant differences were observed between women who sometimes used and who never used contraception, as well as those who planned and did not plan to use contraception, only regarding their ethnicity and level of education. Women who used and/or planned to use contraception were mainly of Serbian ethnicity and had a higher level of education (Table 1). Women who believed that they had good knowledge about contraception more often used all types of contraception except IUD and other contraception methods. Still, this means that they also frequently used natural methods of contraception, which are not recommended due to their low reliability (Table 2).

### 3.6. Correlation Results

A good knowledge level about contraception correlated with both the lifetime use of and the intention to start using contraception. Women who never used contraception assessed their knowledge about contraception as poor. Women who had the intention to start using contraception more often assessed their knowledge about contraception as good. However, women who never used contraception generally did not have the intention to start using contraception after having an induced abortion (Supplementary Table S1).

Women who used condoms also regularly used postcoital contraception, interrupted coitus and counting fertility days. Women who used IUDs also used other methods of contraception. Women who used hormonal contraception also used postcoital contraception and interrupted coitus. Using postcoital contraception positively correlated with interrupted coitus and counting fertility days (Supplementary Table S1).

### 3.7. Regression Results

The adjusted logistic regression model showed that a higher education attainment was the only factor associated with good knowledge about contraception (Table 3). Lifetime contraception being practiced was more likely among women who were of Serbian ethnicity, who had a higher level of education, who had no chronic illnesses and who already had multiple children (Table 4). Serbian ethnicity, having a higher level of education, being a non-smoker and having chronic illnesses was associated with the intention to use contraception after having an induced abortion due to unwanted pregnancy (Table 5).

## 4. Discussion

Almost all women who sought abortion in this study conceived an unwanted pregnancy because they skipped using contraception. This study identified that most women who aborted their pregnancy had used contraception at some point in their lives. While they used a variety of contraception methods, the most common one was the use of condoms. Postcoital contraception, interrupted coitus and the counting of fertile days, although less reliable, were more often practiced compared to using oral contraceptive pills and IUDs. In fact, despite the fact that intrauterine contraception is much more effective than combined oral contraception in preventing repeated abortions, none of the examined women wished to have an IUD inserted immediately after performing their abortion.

A previous qualitative study among Serbian women found that they were often afraid of oral contraceptive pills due to hormonal effects on the body and they perceived the condom as the most reliable contraception method [14]. Contrarily, in Portugal, a country in southern Europe whose female population has a similar size to that of Serbia, women often use oral contraceptive pills and are generally content with that mode of contraception [15]. A systematic review of studies focusing on reasons to avoid using the oral contraceptive pill in Europe, North America and Oceania reported that the underlying reasons are uncomfortable adverse effects which also include changes in libido and mental health but also a desire to be hormone-free [16]. Also, the information on social media regarding the pill can have a negative impact on how women perceive the pill [17] and therefore opt for less reliable methods of contraception, as evidenced in this study. Finally, the use of condoms and oral contraceptive pills require women to be organized with their supply and be mindful and consistent with their use [18], which may be less motivating for some women.

Having knowledge of contraception, choosing a method of family planning specific to an individual and an individual having the agency to use it consistently is necessary to avoid future unwanted pregnancies. In this study, one in five women had never used contraception. Moreover, about one third of women did not intend to use contraception in the future. Unmet contraception needs do not pertain to resource-limited settings. In fact, they have been identified in industrialized countries such as Sweden [19]. Our findings could be explained by the notion that women in this study had little understanding and insufficient knowledge of the biology of conception and fertility as well as contraception possibilities. Women who are ethnic minorities in Serbia in particular may have a limited awareness of family planning methods [20]; this was also corroborated in this study because it was the women from ethnic minorities who were less likely to use contraception post-abortion. Because of this, it is essential to enhance counseling at all levels of health care delivery [21] and especially to not miss the opportunity to deliver counseling after performing an abortion, as it may help to improve women’s health and reduce abortion needs.

In this study, a higher level of education attainment was associated with better knowledge of contraception, lifetime contraception use and the intention to use contraception in the future. These results strongly highlight the need for information dissemination in formal (e.g., school and counseling in health care) and informal settings (peer education) to help women optimize their family planning and overall well-being. Bearing in mind the relevance of education on contraception for women of reproductive age, it is essential to provide reproductive health information to promote the active role of women in the prevention of abortions. In efforts to achieve this, there may be challenges which call for a paradigm shift. For example, some gynecologists in Serbia believe that abortion is an acceptable method of contraception [22,23]. Therefore, to stimulate changes, structural shifts are in place to systematically transform the perception of abortion as a contraception method and implement the prevention of abortions as a priority. Evidence suggests that enhancing confidence in contraception use is the strongest predictor of contraception use in the future [24,25]. Because of this, education on contraceptive methods, their efficacy and their relevance for women’s health could help to reduce complacency, increase awareness and motivate women to choose the prevention of unwanted pregnancies over abortions.

In addition to having a higher level of education and a Serbian ethnicity, women who had the intention to use contraception after this abortion were more likely to be non-smokers and have chronic illnesses. This finding is not surprising, because preventive health behaviors tend to cluster [26]. The prevention of future abortions could be particularly important for women who have chronic illnesses, as the intervention of general anesthesia may carry an increased risk for complications of their condition and, as a result, potentially require multidisciplinary care [27]. Overall, it seems that women who exercise some health behaviors (such as the avoidance of tobacco smoke) and who have more health challenges also have a greater awareness of future contraception. This means that women in other demographic groups, especially ethnic minorities (such as Roma women), should be exposed to contraception counseling.

This study is limited by the fact that data from only one tertiary health center offering abortion care were collected. Moreover, the study’s limitations include its single-center approach and urban focus. The chosen health care center is located in the largest urban area of Serbia. This design provides insights specific to a clinical setting and urban population in Serbia but limits the generalizability of the results to the broader population, including rural areas. Because of this, the results of this study may not apply universally across Serbia and consequently cannot be generalized to all women of childbearing age in the country. Women from the countryside, smaller cities and towns or socio-economically diverse settings may have different practices around contraception and intentions to use contraception after abortion. Additionally, the absence of probabilistic sampling severely limits the external validity of the study’s findings. For the adequate generalization of the results, probabilistic sampling would be required. Self-reported data are personal, especially regarding abortions, which are regarded as controversial. So, this study may also be open to information bias, particularly given the cultural sensitivities surrounding abortion. As our questionnaire did not include any specific response scales and the responses were not uniformly formed for all items, we were not able to estimate the internal consistency by the means of Cronbach’s alpha and McDonald’s omega coefficients. Bearing in mind that the underlying study design was cross-sectional, causal conclusions can be limited. Finally, the questionnaire had a finite number of items and it may not have fully covered all potential covariates. The questionnaire may not have fully captured all relevant factors impacting contraception-related behavior, particularly as the study did not include the opinions of the women’s partners or those of the gynecologists. So, unobserved confounding could be an additional study limitation.

## 5. Conclusions

In conclusion, most women who had an induced abortion due to unwanted pregnancy had used contraception in the past, and most women expressed the intention to start using contraception after their abortion. Therefore, women need to be continuously reminded by their gynecologists of contraception possibilities, especially if they do not desire more children. Moreover, women with chronic illnesses who are motivated to start using contraception should be encouraged to do so, as there are currently numerous contraception methods, and a suitable method for each individual can be defined.

General education is the most important factor contributing to the lifetime use of and intention to use contraception after abortion. Moreover, specific education and counseling regarding reproductive health is needed for all Serbian women to better understand that natural methods of contraception are not advisable due to their low reliability and that only modern methods of contraception have high reliability in the prevention of unwanted pregnancies.

## Figures and Tables

**Table 1:** General and medical characteristics of women who underwent induced abortion.

Characteristics		Lifetime Contraception Use		Between Groups p	Future Plan to Use Contraception		Between Groups p
		Never	Some Time		No	Yes	
Age group	16 to 25	18	66	0.710	22	62	0.482
	26 to 35	37	168		63	142	
	over 35	27	117		45	99	
Nationality	Serbian	71	342	0.001	119	294	0.013
	Roma	8	4		7	5	
	other minorities *	3	5		4	4	
Religion	Orthodox Christian	73	329	0.166	120	282	0.865
	Catholic Christian	1	3		2	2	
	Muslim	8	7		8	7	
	other	0	2		0	2	
	atheist	0	10		0	10	
Education	no or primary	15	26	0.001	18	23	0.004
	secondary	53	205		81	177	
	high	14	120		31	103	
Employment	yes	45	210	0.621	69	186	0.240
	no	33	111		54	90	
	student	4	30		7	27	
Relationship status	single/divorced/widow	6	24	0.720	11	19	0.898
	coupled	11	58		13	56	
	living together	13	34		22	25	
	married	52	235		84	203	
Professional activity	vigorous	15	50	0.470	16	49	0.736
	moderate	44	193		76	161	
	sedentary	23	108		38	93	
Recreation	no	68	281	0.555	111	238	0.100
	yes	14	70		19	65	
Smoking status	smoker	38	156	0.893	67	127	0.047
	ex-smoker	9	58		20	47	
	non-smoker	35	137		43	129	
Smoking amount	≤20 cigarettes	34	156	0.754	67	123	0.087
	>20 cigarettes	13	58		20	51	
Alcohol	no	53	188	0.070	78	163	0.234
	yes	29	163		52	140	
Alcohol frequency	weekly	5	26	0.914	8	23	0.802
	monthly	5	33		10	28	
	rarely	19	104		34	89	
Chronic illnesses	no	74	331	0.179	125	280	0.147
	yes	8	20		5	23	
Gynecological illnesses	no	64	258	0.397	96	226	0.872
	yes	18	93		34	77	
Menstrual cycle	regular	71	304	0.238	118	257	0.369
	not regular	11	47		12	46	
Pregnancies before	no	16	45	0.147	23	38	0.734
	yes	66	306		107	265	
Cesarean section before	no	73	295	0.256	116	252	0.106
	yes	9	56		14	51	
Abortion	first	42	150	0.164	62	130	0.359
	recurrent	40	201		68	173	

Legend: * Hungarian, Slovak, Rusyn, Vlach, Albanian, etc.

**Table 2:** Lifetime contraception use in the overall sample and according to contraception knowledge.

Characteristics		Overall Sample (Frequency)	Knowledge About Contraception		
			Bad (Frequency)	Good (Frequency)	Between Groups p
Condom	no	128	81	47	0.001
	yes	305	84	221	
Intrauterine device	no	418	160	258	0.699
	yes	15	5	10	
Hormonal contraception	no	357	145	212	0.020
	yes	76	20	56	
Postcoital contraception	no	304	125	179	0.048
	yes	129	40	89	
Interrupted coitus	no	305	138	167	0.001
	yes	128	27	101	
Counting fertile days	no	319	137	182	0.001
	yes	114	28	86	
Other contraception	no	430	163	267	0.307
	yes	3	2	1	

**Table 3:** Factors associated with good self-assessed knowledge (classified as good vs. poor) about contraception.

Predictors	Coefficient B	Coefficient Wald	p	OR	Low 95% CI for OR	High 95% CI for OR
Age	0.001	0.003	0.959	1.001	0.963	1.041
Nationality	−0.168	0.236	0.627	0.845	0.429	1.664
Religion	0.103	0.324	0.569	1.108	0.778	1.578
Education	0.182	4.648	0.031	1.199	1.017	1.415
Employment	0.167	0.826	0.364	1.181	0.825	1.693
Salary (EUR)	0.000	0.012	0.912	1.000	0.999	1.001
Relationships	−0.055	0.206	0.650	0.947	0.747	1.200
Professional activity	0.012	0.012	0.913	1.012	0.816	1.255
Recreation	−0.116	0.196	0.658	0.890	0.532	1.490
Smoking	0.003	0.001	0.979	1.003	0.805	1.250
Alcohol	0.032	0.022	0.883	1.032	0.678	1.571
Chronic illness	0.398	0.807	0.369	1.489	0.624	3.552
Gynecologic illness	−0.012	0.002	0.960	0.988	0.617	1.583
Parity	0.002	0.001	0.976	1.002	0.866	1.159
Cesarean section	0.055	0.035	0.852	1.057	0.594	1.880
Recurrent abortion	0.308	1.426	0.232	1.360	0.821	2.254
Constant	−0.283	0.592	0.041	0.753		

Legend: OR—odds ratio; CI—confidence interval.

**Table 4:** Factors associated with lifetime use of contraception.

Predictors	Coefficient B	Coefficient Wald	p	OR	Low 95% CI for OR	High 95% CI for OR
Age	−0.011	0.207	0.649	0.989	0.941	1.038
Nationality	−0.899	7.879	0.005	0.407	0.217	0.762
Religion	0.077	0.108	0.742	1.080	0.683	1.710
Education	0.369	12.910	0.001	1.447	1.183	1.769
Employment	0.253	1.126	0.289	1.288	0.807	2.056
Salary (EUR)	0.000	0.169	0.681	1.000	0.999	1.001
Relationships	−0.072	0.229	0.632	0.931	0.693	1.250
Professional activity	0.001	0.000	0.992	1.001	0.755	1.329
Recreation	0.084	0.058	0.810	1.088	0.548	2.158
Smoking	−0.048	0.115	0.734	0.953	0.720	1.260
Alcohol	0.216	0.601	0.438	1.241	0.719	2.143
Chronic illness	−0.146	2.684	0.041	0.463	0.185	1.163
Gynecologic illness	0.133	0.173	0.677	1.142	0.611	2.132
Parity	0.141	4.143	0.042	1.152	1.005	1.319
Cesarean section	0.404	0.953	0.329	1.498	0.666	3.370
Recurrent abortion	−0.016	0.002	0.963	0.984	0.505	1.918
Constant	0.551	0.809	0.036	1.735		

Legend: OR—odds ratio; CI—confidence interval.

**Table 5:** Factors associated with the intention to start using contraception after induced abortion.

Predictors	Coefficient B	Coefficient Wald	p	OR	Low 95% CI for OR	High 95% CI for OR
Age	−0.035	2.679	0.102	0.966	0.926	1.007
Nationality	−0.864	5.519	0.019	0.422	0.205	0.867
Religion	0.216	1.000	0.317	1.242	0.812	1.898
Education	0.220	4.623	0.032	1.246	1.020	1.522
Employment	−0.132	0.443	0.506	0.876	0.593	1.294
Salary (EUR)	0.000	0.472	0.492	1.000	0.999	1.001
Relationships	−0.013	0.010	0.919	0.987	0.765	1.273
Professional activity	−0.154	1.621	0.203	0.857	0.676	1.087
Recreation	0.371	1.475	0.225	1.449	0.796	2.637
Smoking	0.244	3.935	0.047	1.276	1.003	1.623
Alcohol	0.145	0.387	0.534	1.156	0.732	1.828
Chronic illness	1.188	4.576	0.032	3.282	1.105	9.749
Gynecologic illness	−0.122	0.224	0.636	0.885	0.533	1.469
Parity	0.034	0.174	0.676	1.034	0.882	1.213
Cesarean section	0.539	2.515	0.113	1.715	0.880	3.341
Recurrent abortion	0.217	0.598	0.439	1.242	0.717	2.151
Constant	1.465	1.898	0.048	4.329		

Legend: OR—odds ratio; CI—confidence interval.

## Data Availability

The dataset underlying this study is available upon a reasonable request to the corresponding author.

## Supplementary Materials

The following supporting information can be downloaded at: https://www.mdpi.com/article/10.3390/medicina60121944/s1, Table S1: Correlations of examined parameters regarding contraception use.
