# Supplementary material for: Lifetime Practice and Intention to Use Contraception After Induced Abortion Among Serbian Women in Belgrade
Source: Medicina (Kaunas). 2024 Nov 26;60(12):1944. doi: 10.3390/medicina60121944 (PMC11678184; doi:10.3390/medicina60121944)
Supplement: Supplementary file 1 [file medicina-60-01944-s001.zip › medicina-3266417-supplementary.pdf]

Table S1. Correlations of examined parameters regarding contraception use

Parameters		Knowledge about CC	Lifetime use of CC	Future plan for CC use	Condom	IUD	Hormonal CC	Postcoital CC
Lifetime use of CC	ρ	0.410		0.481	0.746	0.092	0.223	0.315
	p	0.001		0.001	0.001	0.057	0.001	0.001
Future plan for CC use	ρ	0.275	0.481		0.415	-0.041	0.143	0.173
	p	0.001	0.001		0.001	0.392	0.003	0.001
Condom	ρ	0.336	0.746	0.415		-0.043	0.046	0.389
	p	0.001	0.001	0.001		0.368	0.338	0.001
IUD	ρ	0.019	0.092	-0.041	-0.043		0.012	-0.041
	p	0.699	0.057	0.392	0.368		0.800	0.400
Hormonal CC	ρ	0.112	0.223	0.143	0.046	0.012		0.164
	p	0.020	0.001	0.003	0.338	0.800		0.001
Postcoital CC	ρ	0.095	0.315	0.173	0.389	-0.041	0.164	
	p	0.048	0.001	0.001	0.001	0.400	0.001	
Interrupted coitus	ρ	0.227	0.313	0.192	0.253	0.016	0.127	0.165
	p	0.001	0.001	0.001	0.001	0.745	0.008	0.001
Fertility day counting	ρ	0.167	0.289	0.186	0.249	-0.027	0.027	0.127
	p	0.001	0.001	0.001	0.001	0.572	0.569	0.008
Other CC	ρ	-0.049	0.040	0.055	-0.068	0.136	-0.039	-0.054
	p	0.308	0.402	0.256	0.158	0.004	0.424	0.259

Legend: CC – contraception, IUD – Intrauterine devise
